# Realistic fault detection of li-ion battery via dynamical deep learning

**DOI:** 10.1038/s41467-023-41226-5

**Published:** 2023-09-23

**Authors:** Jingzhao Zhang, Yanan Wang, Benben Jiang, Haowei He, Shaobo Huang, Chen Wang, Yang Zhang, Xuebing Han, Dongxu Guo, Guannan He, Minggao Ouyang

**Affiliations:** 1 IIIS Tsinghua University, Beijing, China; 2 Shanghai Qizhi Institute, Shanghai, China; 3 State Key Laboratory of Intelligent Green Vehicle and Mobility, School of Vehicle and Mobility, Tsinghua University, Beijing, China; 4 Department of Automation, Beijing National Research Center for Information Science and Technology, Tsinghua University, Beijing, China; 5 Beijing Circue Energy Technology Co. Ltd., Beijing, China; 6 School of Automation Science and Electrical Engineering, Beihang University, Beijing, China; 7 Department of Industrial Engineering and Management, College of Engineering, Peking University, Beijing, China; 8 National Engineering Laboratory for Big Data Analysis and Applications, Peking University, Beijing, China; 9 Peking University Changsha Institute for Computing and Digital Economy, Changsha, China

**Keywords:** Batteries, Computer science, Batteries

## Abstract

Accurate evaluation of Li-ion battery (LiB) safety conditions can reduce unexpected cell failures, facilitate battery deployment, and promote low-carbon economies. Despite the recent progress in artificial intelligence, anomaly detection methods are not customized for or validated in realistic battery settings due to the complex failure mechanisms and the lack of real-world testing frameworks with large-scale datasets. Here, we develop a realistic deep-learning framework for electric vehicle (EV) LiB anomaly detection. It features a dynamical autoencoder tailored for dynamical systems and configured by social and financial factors. We test our detection algorithm on released datasets comprising over 690,000 LiB charging snippets from 347 EVs. Our model overcomes the limitations of state-of-the-art fault detection models, including deep learning ones. Moreover, it reduces the expected direct EV battery fault and inspection costs. Our work highlights the potential of deep learning in improving LiB safety and the significance of social and financial information in designing deep learning models.

## Abstract (web summary)

Accurate evaluation of Li-ion battery safety conditions can reduce unexpected cell failures. Here, authors present a large-scale electric vehicle charging dataset for benchmarking existing algorithms, and develop a deep learning algorithm for detecting Li-ion battery faults.

## Introduction

Achieving net-zero emissions entails transportation electrification^1,2^ and decarbonization^3^. Electric vehicles (EVs) with lithium-ion batteries (LiBs) are the most widely adopted devices due to their rapid performance improvements and cost reductions^4–6^. One major concern to EV owners and manufacturers is battery safety^7,8^. EV fires last longer and are more unpredictable, requiring frequent costly inspections for EV manufacturers. Therefore, early prediction of battery failure events could save significant social costs and promote EV adoption. However, as EV batteries are highly complex nonlinear systems, designing algorithms that understand the failure mechanisms—including short circuit, physical damage, overcharge/overdischarge, thermal abuse, etc.^9–12^—remains challenging.

Existing studies on battery safety have explored both physics-based^13–17^ and data-driven^18–25^ approaches to address issues caused by defective battery cells. However, applying these methods in real life still has a long way to go for two reasons. First, existing algorithms require further testing, as validations are only done in small-scale experimental/lab settings. In contrast, the success of data science in many other fields, such as video games, vision, translation and protein structure predictions, were developed and evaluated on large-scale, real-world datasets^26–29^. Second, many existing algorithms rely on information that is unavailable in real-world settings. Designing an EV battery fault detection algorithm that is implementable and effective for both EV manufacturers and owners needs to take practical social factors into account^30,31^, such as the data availability, economic trade-offs, sensor noise, and model privacy. In short, existing studies do not reveal the power of deep learning for EV battery fault detection with large-scale publicly available EV charging datasets, nor do they discover how practical factors should inform algorithm design and deployment.

In this work, we release three EV charging datasets with over 690,000 charging snippets from 347 EVs. Our datasets enable us to benchmark known deep learning models and compare them against the more conventional data-driven approaches. We found that although all models can achieve nontrivial detection power (as measured by accuracy and recall) by learning from the data, their performances could incur high economic costs, which are around 10^3^ Chinese Yuan (CNY) per vehicle according to our computation.

To address the problem, we further develop a deep learning model termed dynamical autoencoder for anomaly detection (DyAD) with a privacy-friendly and communication-efficient design. Our proposed model is the first deep learning model tailored for large-scale real-world EV LiBs data. It exploits the hidden Markov model of battery data in designing the neural network training pipeline and balances EV LiB accident costs against inspection costs based on empirical statistics. Our model differs from existing deep anomaly detection models in two aspects. First, it adopts a dynamical system formulation and partitions the data features into system inputs and system responses. It then detects the abnormality in the input-to-response mappings. In contrast, most existing deep learning algorithms for fault detection treat each dimension of the data features equally and is not tailored for dynamical system with external inputs. Second, our model exploits the structure in EV fault labels by bridging the predictions between the vehicle system level and the LiB charging snippet level with a robust scoring procedure. We test our proposed algorithm on the three datasets with 55 abnormal vehicles (vehicles with LiB fault) and 292 normal vehicles (vehicles without LiB fault). Compared with conventional physics-based methods and the state-of-the-art deep learning models, our algorithm produces a dominating average receiver operating characteristic (ROC) curve for predicting LiB anomalies, and it lowers the overall costs (33–50% savings) of EV battery accidents and inspections. This work highlights the effectiveness of deep-learning algorithms in predicting EV LiB faults with limited anomaly samples and the practicality of our algorithm design.

## Results

### Challenges in real-world EV battery fault detection

**Fig. 1:**
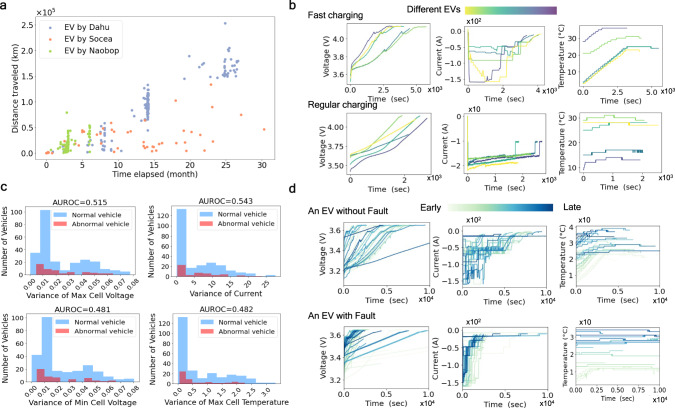
EV dataset and challenges in fault detection. **a** The data used in this study contains vehicles from three manufacturers, aliased Dahu, Socea and Naobop. Each dot represents the amount of data between the first charging record and the last charging record of a single vehicle. The *x*-value and *y*-value of a dot indicate the distance traveled and the time elapsed in records. **b** Sampled charging segments show that real-world charging patterns are diverse and irregular. Charging modes can be categorized into fast charging and regular charging based on the level of the current. **c** Normal and abnormal EVs are poorly differentiated with canonical features such as the variance in cell voltage, current, or temperature, as an AUROC (area under receiver operating characteristic) around 0.5 can be achieved by random guesses. Higher AUROC value indicates greater prediction power. **d** A detailed view of LiB charging snippets from two sampled EVs. The comparison suggests that there is no simple feature to detect EV fault.

Real-world anomaly detection models can only make use of observational data from existing battery management systems (BMSs). To facilitate model development, we release three large-scale datasets collected from the EV data platform hosted by Tsinghua University. Vehicles in each of our three released datasets are of the same make. We generate a random code name for each manufacturer and summarize the data statistics in Fig. 1a. Each charging snippet of a vehicle stores current, voltage and temperature as time series (Fig. 1b). Vehicle-level fault labels are generated from drivers’ reports and confirmed by engineers based on the identification of lithium plating, low electric range, over-high temperature or unexpected voltage changes (too low, inconsistent among cells, etc.). These labels are created case-by-case and cannot be described by rule-based expressions on data. The abnormal data at or near battery failures are removed so that successful predictive models need to identify battery problems at least days ahead based on historical data. They may also be used for tasks beyond anomaly detection such as battery capacity degradation prediction.

Given the available data, few known fault detection algorithms can be directly applied due to the limited available information of the vehicle. Indeed, in realistic setups, model parameters such as open circuit voltage or internal resistance are very often missing. Hence, one common approach to fault detection is based on the variance within temperature or voltage. However, in Fig. 1c, we see that such approach provides weak predictions. In Fig. 1d, we further plot the variations of charging records across time for one normal vehicle and one vehicle with fault. The plots confirm that fault detection based on simple features or variation analysis is difficult because of the complex fault mechanism. To address this problem, we propose a practical deep learning framework customized for large-scale LiB fault detection.

### A deep learning framework with a dynamical autoencoder

**Fig. 2:**
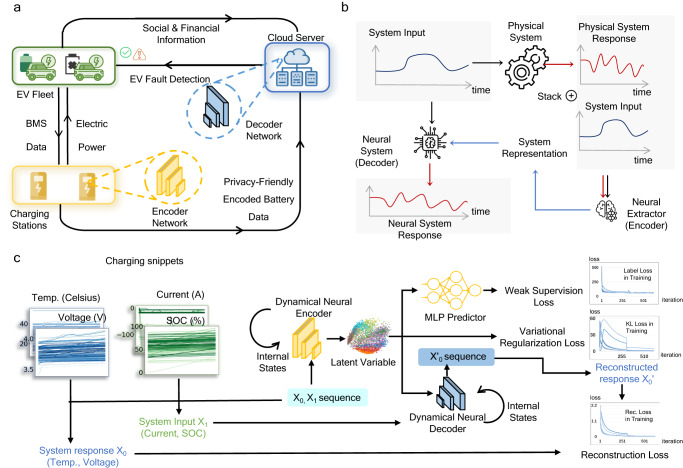
Illustrations of the model deployment and the dynamical autoencoder model. **a** The model deployment involves communication among three parties: the charging station, the EV fleet and the cloud server. The charging stations first collect BMS data and transmit encoded privacy-friendly battery data to the cloud server. The cloud server generates a fault score via reconstruction. The cloud detector then computes the economically optimal prediction based on social and financial statistics from EV data platforms. **b** The encoder in our model does not encode the system inputs/outputs, but encodes the mapping between the system inputs and outputs. The decoder reflects the physical system. It translate the system input into the system response based on the parameters generated by the encoder. **c** An illustration of the neural network training procedure. The input BMS data are split into two groups: system input (SOC and current) and system response(voltage and temperature). The battery, as the dynamical system, takes in the system input and generates the system response. The model parameters are updated by minimizing a total training loss consisting of three data pipelines, corresponding to the encoder-decoder reconstruction, the mileage supervision and the Kullback-Leibler (KL) regularization.

Our deep learning framework is designed to be compatible with real-world deployment, as in Fig. 2a. The deep learning model features an encoder-decoder structure and trains on EV BMS data without requiring additional sensors. Social and financial statistics such as fault and inspection costs, fault rate are used to optimally configure the fault detection model for the best economic performance. After training, our model can be deployed in a two-way privacy-preserving manner due to the encoder-decoder structure^32,33^. The encoder network is deployed at charging stations, whereas the fault detection module is cloud-based. Such a deployment design brings threefold advantages: (i) a service producer can maintain possession of their model details and thus avoid adversarial attacks or model leakage; (ii) EV customers preserve sensitive information such as mileage, charging time, and location; (iii) data communication is reduced by sending over encoded partial data.

Our framework and datasets enable us to adopt and train the latest anomaly detection models. However, we found that while several existing deep models can be adapted to our datasets of EV charging snippets, they fail to exploit the EV LiB dataset structure for the two challenges below, to which we strategically designed our deep learning model, respectively.

First, existing deep learning algorithms detect anomalies by learning a distribution over the dataset and reject infrequent data points. However, such logic could falsely detect a normal battery (LiB without any faults) if the battery is charged with a rare current pattern (specific charging state with a certain current). To address this issue, we propose the dynamical autoencoder for anomaly detection customized for anomaly detection on dynamical systems. Our model also adopts an encoder-decoder structure (see Fig. 2b) but the decoder, rather than constructing the entire data from the latent variables, now decodes the system response from the system input (see Supplementary Fig. 1 for an example). This intuition can be formalized by viewing the LiB data as collected from random nonlinear dynamical systems described by hidden Markov models (see Supplementary Note 1). Exploiting this structure customizes the general deep learning model for the LiB data. The dynamical autoencoder model is shown in Fig. 2c. The system control inputs include state of charge (SOC) and current, and system response include voltage and temperature. The encoder maps inputs (SOC, current) and outputs (voltage, temperature) into the latent variables that represent system parameters. Our model then detect system anomaly based on the discrepancy between the reconstructed and the observed system responses. In addition, auxiliary losses are added to achieve latent space regularization as in variational autoencoder and to provide weak supervision via mileage data (adding vehicle range as input).

A second problem for EV LiB data is that abnormal vehicle labels are sparse and made at the vehicle level; therefore, the labels may not truthfully reflect the condition of each data point corresponding to a particular charging snippet. To address this, we developed a robust scoring procedure (see Methods) to generate vehicle-level predictions from charging snippet predictions. The designed robust scoring procedure is parametrized by two hyperparameters *τ* and *p*, which predicts whether a charging snippet is abnormal by thresholding the reconstruction error at value *τ* and then predicts whether a vehicle is abnormal by averaging the top *p* percentile errors. The training pipeline is summarized in Fig. 2c.

### Comparison against state-of-the-art algorithms

**Fig. 3:**
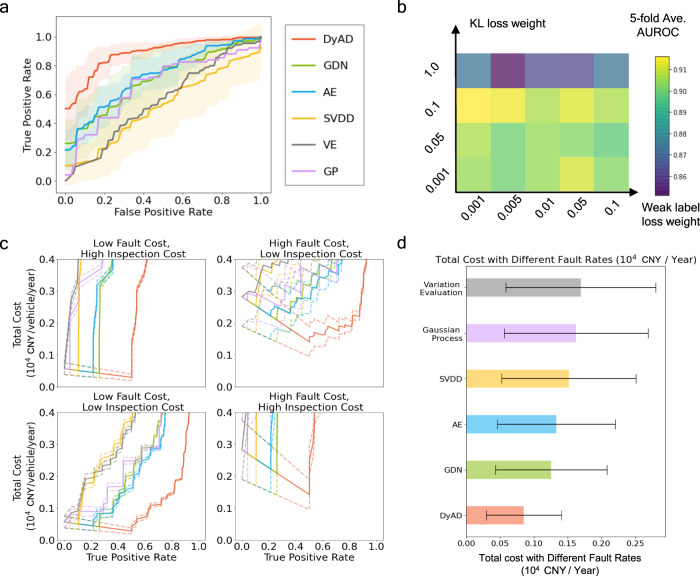
Evaluating prediction accuracy and EV battery cost. **a** The average ROC curves for the five algorithms. The solid curves indicate the average values out of five cross validation runs, and the shaded regions indicate the standard deviations of the trials. Our proposed procedure (orange) significantly outperforms other deep learning baselines (green, blue, yellow) and the standard variation evaluation method (gray). **b** The five-fold average AUROC of our proposed DyAD with different weights of the auxiliary training losses (KL regularization and mileage label loss). The variation of the average AUROC indicates that the auxiliary training losses can nontrivially improve our model performance. The cost ranges can be found in Methods. **c** The sum of direct fault cost and inspection cost for EV batteries based on the statistics (battery fault rate, battery fault cost and vehicle inspection cost) we collected from an EV platform. The horizontal axis indicates the true positive rate achieved by each model for the corresponding cost value. **d** The minimum cost achieved for each algorithm by optimizing the total cost against the true positive rate. The confidence range is evaluated from our estimation of the vehicle fault rate (from 0.038% to 0.075%).

We compare our proposed algorithm against the widely used baselines including the graph deviation network (GDN), vanilla autoencoder (AE), support vector data description (SVDD), Gaussian process model (GP) and a data-driven battery fault detection algorithm (variation evaluation, VE) on the released datasets. The implementation details of the methods are provided in the Supplementary Note 2 and Supplementary Fig. 5. The detection performance, which is measured by the area under receiver operating curve (AUROC) based on the true positive rate and false positive rate, is provided in Fig. 3 and Table 1. The results show that the proposed dynamical autoencoder approach achieves the best detection results by a 16–33% AUROC boost (Fig. 3a) and a smaller variance compared to other algorithms (Table 1). The results also show that the auxiliary loss can further improve the detection performance (Fig. 3b). In addition, as depicted in Supplementary Fig. 2, the charging snippets from a normal vehicle and a faulty vehicle are rather difficult to be differentiated and are frequently falsely classified by the baselines. In contrast, the dynamical formulation in dynamical autoencoder can correctly pinpoint the charging snippets from both the normal and faulty vehicles.

**Table 1:** Mean and standard variance of AUROC (%) values of considered algorithms

Algorithm	AUROC (%)	Average Direct Cost (10^4^ CNY)
**DyAD**	**88.6** ± **2.9**	**0.085**
GDN	70.3 ± 5.5	0.126
AE	72.8 ± 13.4	0.133
SVDD	51.5 ± 8.26	0.152
GP	66.6	0.162
VE	55.6	0.169

Our proposed method is highlighted in bold. The average direct costs of fault and inspection are in the unit of 10^4^ CNY/vehicle/year and calculated using the mean of the cost ranges. The VE and GP method has little internal randomness and hence no variance is reported.

The enhanced detection performance of the proposed dynamical autoencoder approach can lead to large economic advantages. We obtain the ranges of LiB fault rate based on the reported battery incidents provided to Tsinghua by EV manufacturers which we cannot disclose due to anonymization requests. The fault rate is estimated from 1.2 million EVs across major cities including Beijing, Shanghai, Guangzhou and Shenzhen. We further estimate fault cost, and inspection cost estimated from Circue’s data obtained via partnership with insurance companies in China. Based on the average fault rate, the cost information and the ROC curves, we compute the expected direct costs of LiB fault and inspection (see Methods), as shown in Fig. 3c. The results show that optimally configuring the algorithm along the ROC curve has significant impacts on the overall costs. For all deep learning algorithms (DyAD, GDN, AE, and SVDD), the expected direct costs first decrease and then increase. The best true positive rate of DyAD is ~50%. The minimum cost of the six algorithms (DyAD, GDN, AE, SVDD, GP and VE) is also provided by optimizing the true positive rate, as shown in Fig. 3d. In average, our DyAD reduces the expected direct costs by 33% when compared with the state-of-the-art deep learning algorithms, and 50% when compared with VE, a non-deep-learning method.

In high fault rate scenarios, the inspection scale would have an impact on the reputation of the EV manufacturer and in turn the sales revenues, which we do not yet have solid data to analyze. Such social and financial values (fault rate, fault cost, direct and indirect inspection costs etc.) vary by regions, and we are not trying to provide an accurate model configuration for every region or company here. Rather, we highlight the significance of such practical factors in algorithm configuration, as proposed in our deep learning framework (Fig. 2).

### Knowledge learned by the dynamical autoencoder model

**Fig. 4:**
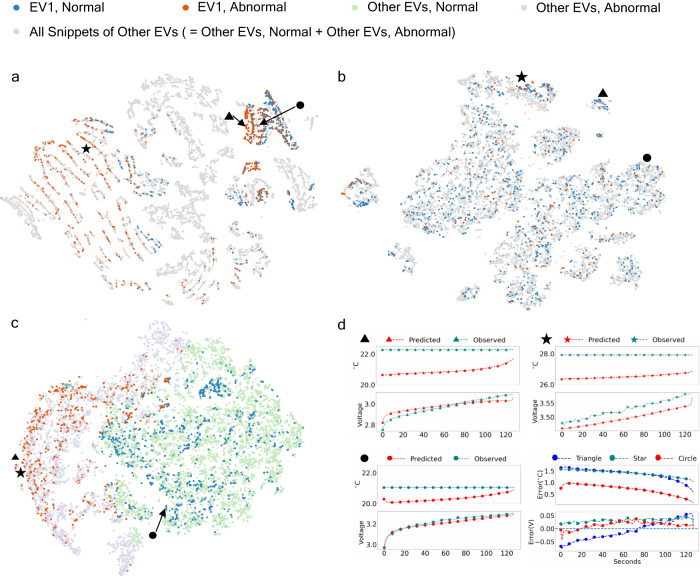
The evolution of embeddings of all fifteen abnormal vehicles in Dataset Dahu from the input layer to the output layer of the dynamical autoencoder model with t-distributed stochastic neighbor embedding visualization. Each subplot visualizes the dimension-reduced data of (**a**) the data input, (**b**) the inferred latent variable, and (**c**) the reconstructed observation. All snippets are identified as either abnormal or normal ones, marked as purple or green points in the output layer, respectively. One EV (named EV1) is highlighted. Its snippets are marked as red and blue points according to their abnormality determined by the dynamical autoencoder model. The predicted and observed curves of the three snippets (marked as ▴, ⋆, and •) selected from EV1 include two dimensions: maximum battery temperature, and minimum cell voltage. **d** The predicted, observed, and corresponding error curves of three charging snippets marked as ▴, ⋆, and •.

A more transparent interpretation of the model prediction can provide advice for EV maintenance or even guide the LiB manufacturing process. To this end, we interpret the internal mechanism of the dynamical autoencoder model by visualizing the distributions of embedded snippets at three different layers: the input layer, the latent layer between the encoder and the decoder, and the output layer computed as the difference between the prediction and the observation. To this end, we visualize how the neural network activation values for each data point (a charging snippet in a vehicle) change across different layers of the deep model. The activation values serve as a high level representation for each data, but is high-dimensional. Therefore, we use t-distributed stochastic neighbor embedding, a nonlinear dimension reduction technique, to project each charging snippet onto a 2D plane for each layer, as shown in Fig. 4.

More specifically, we choose an arbitrary vehicle and highlight the dimension-reduced representations of its LiB charging snippets in red and blue (indicating that the snippets are abnormal and normal, respectively), as in Fig. 4a–c. Snippets of other vehicles are colored gray. In Fig. 4c, the same red and blue points are plotted at the output layer, and other data points are further labeled based on whether they are normal (green) or not (purple). Data points that are closer together has similar semantic meaning to the neural network. We observed that while the snippets of different colors are jumbled in the input and latent layers (Fig. 4a, b), the deep model can successfully separate normal snippets from abnormal snippets in the output layer (Fig. 4c).

To illustrate the above observation with a more concrete example, three LiB charging snippets are picked and marked as ▴, ⋆, and •. In Fig. 4d, we plot the predicted and observed curves of these three data points. In particular, two quantities, the LiB temperature and voltage, are presented to illustrate the errors between the predicted and observed curves. As shown in the right bottom figure of Fig. 4d, the two abnormal snippets (▴, ⋆) have larger prediction errors. The embeddings of the data points are labeled in Fig. 4a–c. We note that the abnormal snippets are far apart in the input and latent layers, but become adjacent in the last output layer, which indicates that the designed prediction errors are good features for clustering abnormal snippets. Hence, based on the prediction errors, our DyAD model can determine an adaptive threshold intelligently, filter the abnormal snippets by ranking reconstruction errors, and accurately detect potential battery faults.

## Discussion

In summary, the research work presented here aims to address the LiB fault detection problem by proposing a realistic deep learning pipeline and releasing a large-scale EV fault datasets with more than 690,000 charging snippets. Our model runs on existing BMS data, and can be readily deployed in real-world settings. Our model adopts dynamical formulation and robust scoring, and can improve the detection performance upon existing procedures. It also reduces the expected direct fault and inspection costs by 33–50%.

We highlighted the role of social and financial statistics, specifically the fault rate and the direct costs of EV battery fault and inspection, in configuring our deep learning model. Such statistics may vary by region, battery chemistry and manufacturer, thus the learning framework should be adaptive to the heterogeneity in these factors. While indirect costs may be hard to accurately capture, they are functions of the base fault rate, inspection scale, market share, etc. Each EV company might strategically project the indirect costs into their models and update them dynamically.

The data privacy of the EV owner and manufacturer is central to the large-scale application of EV fault detection. By deploying encoders and decoders separately at EV charging stations and the cloud, data are automatically encrypted and privacy-friendly. In addition to consumer data protection, this deployment structure also enables EV manufacturers to cooperatively train the encoder without direct data sharing and design a decoder that is adaptive to their own social and financial statistics.

We believe that our dataset and pipeline can serve as a first step for the artificial intelligence and the energy communities to jointly address the safety of EVs, especially given that many problems remain unsolved. One open ended and important problem is model interpretation. A more transparent interpretation in the language provided by battery mechanism research could provide advice for EV maintenance or even guide the battery manufacturing process. For example, besides SOC, current, voltage, and temperature used in this work, the dynamics of more physics-informed battery parameters, such as capacity and internal resistance, could be estimated^34^ or embedded in our model for battery safety with real-world data. Another challenging problem is to quantify the forecast horizon, as it would be beneficial to know for how long the model can predict battery problems in advance. We show in Supplementary Figs. 3 and  4 that the anomalies were detected through out the records, and hence it is difficult to determine the forecast horizon. Moreover, while this work has focused on the application of dynamical deep learning with robust scoring for EV LiB fault detection, such deep learning framework is promising for other fault detection tasks, particularly those for dynamical systems such as photovoltaic panels^35^, robotic navigation^36^, water treatment physical test-bed systems^37^, and spacecraft^38^.

## Methods

### Dynamical autoencoder

We provide more details on applying the dynamical autoencoder model to detecting battery anomalies. The dynamical autoencoder contains three groups of parameters: the parameters for the encoder *θ*, the parameters for the decoder *ζ* and the parameters for the multiperceptron head *ξ*. The encoder and the decoder are parameterized by GCN networks^39^. All parameters are optimized with minibatch stochastic gradients to reduce a total training loss with three components: the reconstruction loss, the regularization loss and the mileage supervision loss, as illustrated below,

1 $$l(\xi,\, \zeta,\,\theta )={l}_{{{{{\rm{recon.}}}}}}(\zeta,\, \theta )+{l}_{{{{{\rm{reg.}}}}}}(\theta )+{l}_{{{{{\rm{mileage}}}}}}(\xi,\, \theta ).$$

More formally, the reconstruction loss for a single data snippet (*x*_0_, *x*_1_) is defined as

2 $${l}_{{{{{\rm{recon.}}}}}}(\theta,\, \zeta,\, {x}_{0},\, {x}_{1})={{\mbox{MSE}}}({{{\mbox{Decoder}}}}_{\theta }(z,\,{x}_{0}),\,{x}_{1}),{{\mbox{where}}}\,\,z={{{\mbox{Encoder}}}}_{\zeta }({x}_{0},\,{x}_{1}).$$

In the above equation, *x*_0_ denotes the input signal (i.e., the current and the SOC), *x*_1_ denotes the system response (e.g., min voltage, max voltage, average voltage, min temperature and max temperature), *z* denotes the latent variable that represents the state of the dynamical system and MSE stands for the mean squared error.

In addition, as in the case for variational autoencoders^40^, KL regularization is used to the latent space to avoid overfitting^41^,

3 $${l}_{{{{{\rm{reg.}}}}}}(\theta,\, {x}_{0},\, {x}_{1})=\parallel {z}_{\mu }{\parallel }^{2}+tr({z}_{\sigma }^{2})-\log (| {z}_{\sigma }^{2}| ),\, {{\mbox{where}}}\,\,z={{{\mbox{Encoder}}}}_{\zeta }({x}_{0},\, {x}_{1}):=[{z}_{\mu },\, {z}_{\sigma }].$$

Note that the latent variable is partitioned into [*z*_*μ*_, *z*_*σ*_] and represents the parameters of the distribution of the random dynamical system. We refer the reader to the supplementary material for a more detailed discussion.

Last, a mileage supervision is also added to guide learning.

4 $${l}_{{{{{\rm{mileage}}}}}}	(\theta,\,,\, {x}_{0},\, {x}_{1})={{\mbox{MSE}}}({{{\mbox{MLP}}}}_{\xi }({{{\mbox{Decoder}}}}_{\theta }(z,\, {x}_{0})),\, {{\mbox{mileage}}}),\, \\ 	 {{\mbox{where}}}\,\,z={{{\mbox{Encoder}}}}_{\zeta }\, ({x}_{0},\, {x}_{1}).$$

The impact of the mileage supervision is illustrated in Fig. 3b and it shows that asking the encoder to keep the mileage information can boost performance.

In our proposed deep learning pipeline, the encoder and decoder models adopt the GRU^42^ model with three layers and 32 hidden dimensions. The latent space between the encoder and decoder has 32 dimensions. The model is trained with the Adam^43^ optimizer, in which the learning rate is set to be 0.001 and each minibatch contains 128 samples.

#### Robust scoring technique

In particular, we predict whether a charging snippet is abnormal by thresholding the reconstruction error at value *τ* and then predict whether a vehicle is abnormal by averaging the top *p* percentile errors. Both *τ* and *p* are finetuned on the training dataset. In particular,

5 $$	{L}_{{{{{\rm{vehicle}}}}}}=({l}_{1}(\theta,\, \zeta,\, {x}_{0},\, {x}_{1}),\, ...,\, {l}_{m}(\theta,\,\zeta,\,{x}_{0},\, {x}_{1})),\, {{\mbox{where}}}\,\,m=\,{{\mbox{number of snippets}}}\,,\\ 	 \,{{\mbox{Vehicle Error}}}\,=\left(\mathop{\sum }\limits_{i=1}^{n}{L}_{{{{{\rm{vehicle}}}}}}(i)\right)/n,\, {{\mbox{where}}}\,\,n=p\times m.\\ 	 \,{{\mbox{A vehicle is abnormal}}},\, {{\mbox{if}}}\,\,\,{{\mbox{Vehicle Error}}}\, \, > \, \tau .$$

*L*_vehicle_ denotes all the reconstruction loss of snippets ranking in one vehicle, *m* denotes the amount of snippets of this vehicle, and Vehicle Error denotes the averaged reconstruction error in the top *p* percentile.

#### Expected direct cost calculation

The expected direct costs of EV battery fault and inspection are calculated as:

6 $$y(p,\, {c}_{{{{{\rm{f}}}}}},\, {c}_{{{{{\rm{r}}}}}},\, {q}_{{{{{\rm{TP}}}}}},\, {q}_{{{{{\rm{FP}}}}}})=p(1-{q}_{{{{{\rm{TP}}}}}}){c}_{{{{{\rm{f}}}}}}+[\,p{q}_{{{{{\rm{TP}}}}}}+(1-p){q}_{{{{{\rm{FP}}}}}}]{c}_{{{{{\rm{r}}}}}}$$

where, *p* is the fault rate of an EV battery; *c*_f_ is the direct cost of an EV battery fault; *c*_r_ is the direct cost of EV battery inspection; *q*_TP_ is the true positive rate of an EV battery fault detection algorithm; and *q*_FP_ is the false positive rate.

According to information from EV battery monitors/operators, the EV battery fault rate *p* ranges from 0.038% to 0.075%; the direct cost of an EV battery fault *c*_f_ ranges from 1 to 5 million CNY per vehicle; and the direct cost of an EV battery inspection *c*_r_ ranges from 8 to 55 thousand CNY per vehicle. We use the lower and upper bounds of the above ranges to calculate the expected direct costs in scenarios with high/low fault rates, fault costs, and inspection costs, respectively.

### Supplementary information

Supplementary Information

Peer review file

## Data Availability

The raw EV data are protected and are not available due to data privacy laws. The processed EV data are available with 10.6084/m9.figshare.23659323 at the link https://figshare.com/articles/dataset/Realistic_fault_detection_of_Li-ion_battery_via_dynamical_deep_learning_approach/23659323.

## Code availability

The code can be downloaded from https://github.com/962086838/Battery_fault_detection_NC_github. We also provide an additional backup for both the code and the data together at https://disk.pku.edu.cn:443/link/37D733DF405D8D7998B8F57E4487515A.
