# Supplementary material for: Realistic fault detection of li-ion battery via dynamical deep learning
Source: Nat Commun. 2023 Sep 23;14:5940. doi: 10.1038/s41467-023-41226-5 (PMC10517941; doi:10.1038/s41467-023-41226-5)
Supplement: Supplementary file 1 — Supplementary Information [file 41467_2023_41226_MOESM1_ESM.pdf]

Supplementary Information: Realistic Fault Detection of Li-ion Battery via Dynamical Deep Learning

Jingzhao Zhang[1,2+], Yanan Wang[3,+], Benben Jiang[4,+], Haowei He[1], Shaobo Huang[5], Chen Wang[6], Yang Zhang[5], Xuebing Han[3], Dongxu Guo[3], Guannan He[7,8,*], and Minggao Ouyang[3,*]

[1]IIIS, Tsinghua University
[2]Shanghai Qizhi Institute.
[3]State Key Laboratory of Intelligent Green Vehicle and Mobility, School of Vehicle and Mobility, Tsinghua University.
[4]Department of Automation, Beijing National Research Center for Information Science and Technology, Tsinghua University.
[5]Beijing Circue Energy Technology Co., Ltd.
[6]School of Automation Science and Electrical Engineering, Beihang University.
[7]Department of Industrial Engineering and Management, College of Engineering, Peking University.
[8]National Engineering Laboratory for Big Data Analysis and Applications, Peking University.
[+]These authors contributed equally: Jingzhao Zhang, Yanan Wang, Benben Jiang
[*]email: gnhe@pku.edu.cn, ouymg@tsinghua.edu.cn.

Supplementary Figure 1: Training example. Supplementary Figure 1 indicates that after training, our neural network model successfully learns the battery dynamics and can accurately predict the voltage and temperature given a charging pattern.

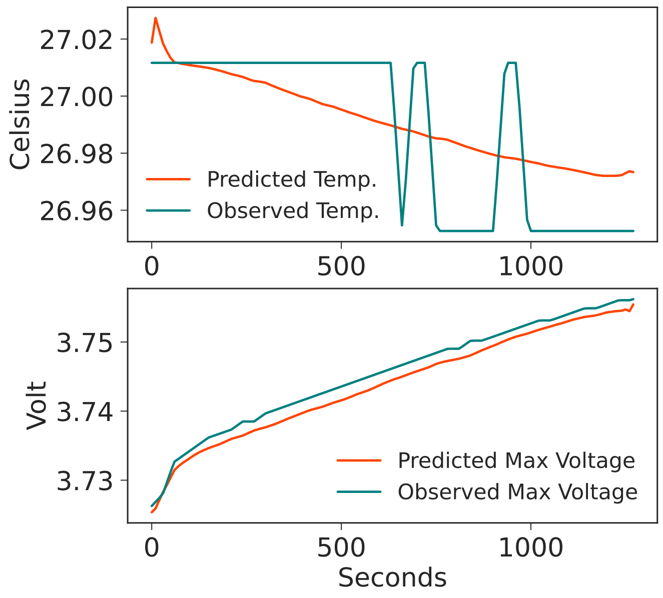

Supplementary Figure 2: Examples of misclassified EV charging records. We provide commonly misclassified EV charging segments below. a. Charging snippets from a normal vehicle that is frequently falsely detected by baselines, but not by the DyAD model. b. Charging snippets from a vehicle with fault that is frequently falsely ignored by baselines, but not by the DyAD model.These samples are correctly classified by DyAD.

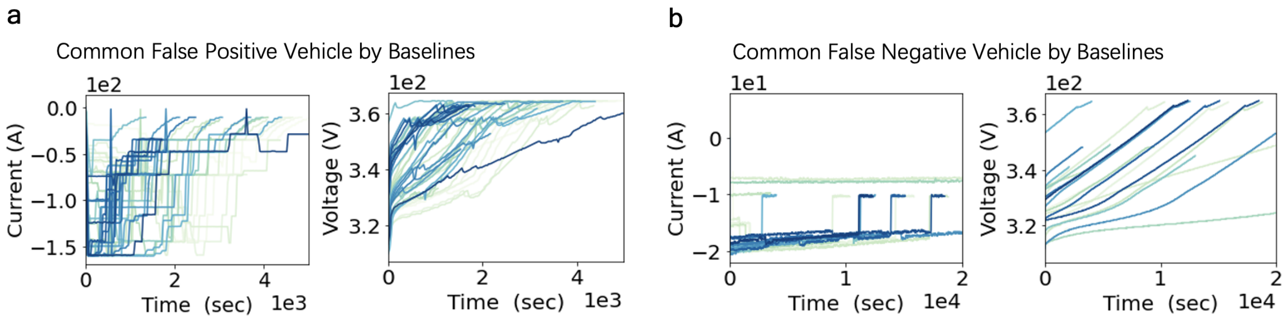

Supplementary Figure 3: The distribution of abnormal labels by DyAD. We provide the detected snippets of one of the abnormal EVs (EV1 in Figure 4) with 234 snippets (a). Among the detected snippets, two snippets in different positions are selected (snippets No.63 and No.109) and plotted in subplots (b)(c), demonstrating that the positive detected frequency is related to the increasing voltage variation of the abnormal cell to the other normal cells.

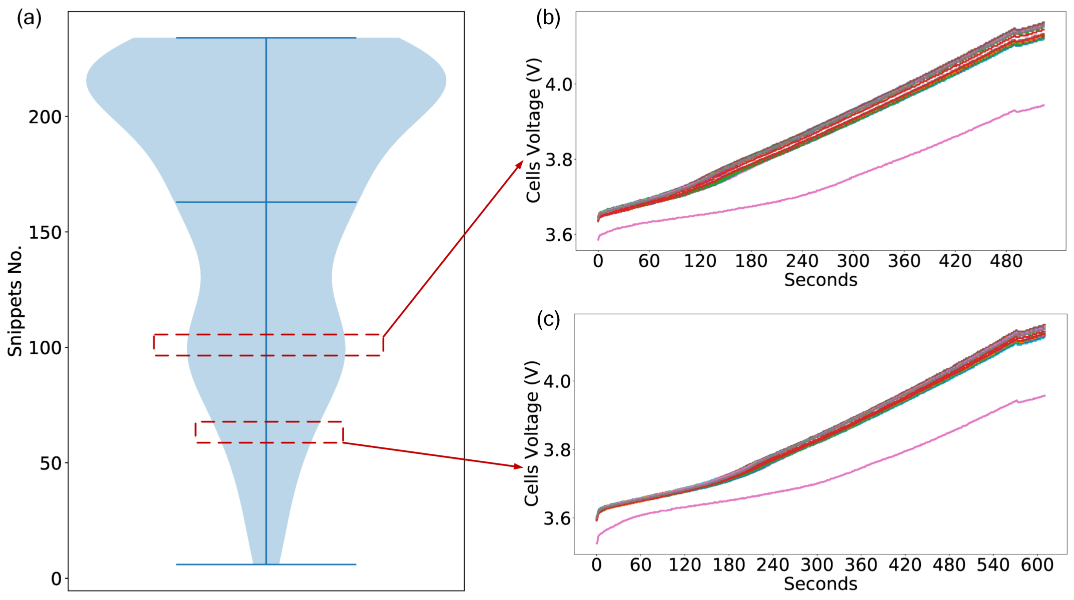

Supplementary Figure 4: Detected snippets of abnormal EVs by DyAD. We provide the distributions of the detected snippets of all fifteen abnormal EVs for training in the first dataset (Dataset Dahu) as violin plots.

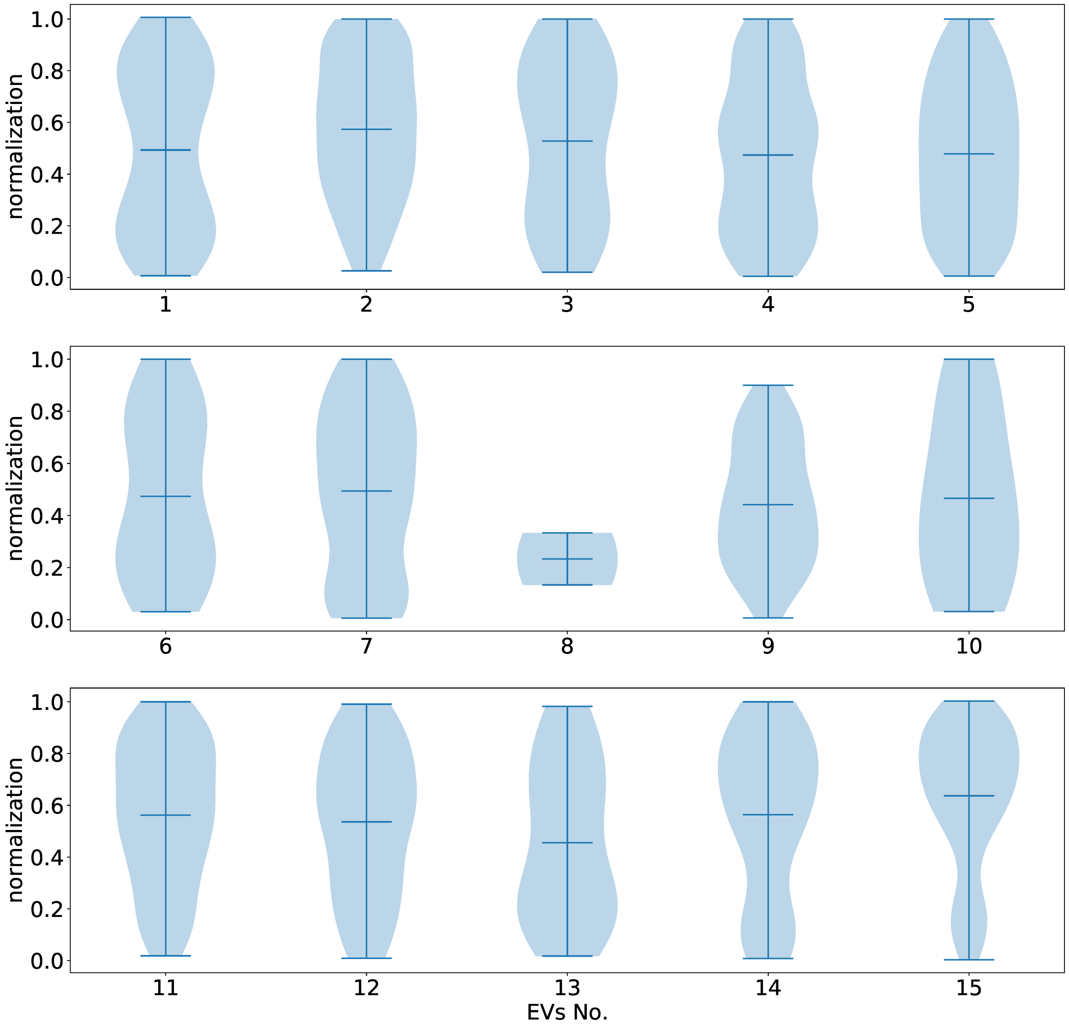

Supplementary Figure 5: Variation evaluation method. Variation evaluation (VE) is a non-deep-learning baseline. According to our experiments summarized in Table 1, it achieves comparable performance to off-the-shelf deep learning algorithms in battery anomaly detection. The process of the variation evaluation (VE) method[1] is illustrated here. In the VE method, five indicators in the charging snippets of EVs, that is, voltage variation, temperature variation, resistance variation, capacity variation, and electric quantity variation, are applied to evaluate cell-to-cell variations for further battery fault detection or cell balance. From the statistic aspect, the VE method firstly calculates the root-mean-square error (RMSE), the variation coefficient, the standard deviation, and the range variation of the five indicators. With predetermined reasonable thresholds, the VE method obtains an unweighted score for every part of the five indicators, then allocates suitable weight factors for the five indicators by analytic hierarchy process (AHP), to calculate the corresponding weighted scores. Finally, the VE method quantizes the cell-to-cell variation into a total score with the weighted scores of the five indicators.

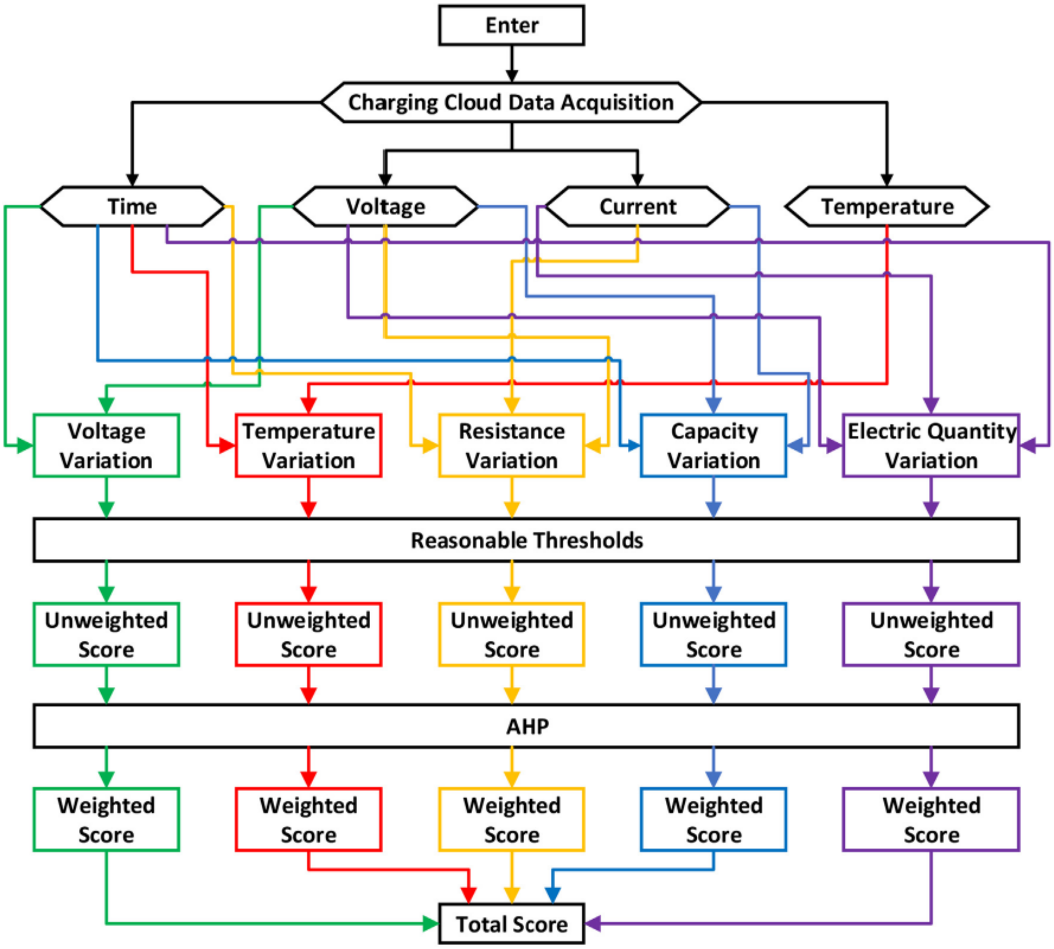

Supplementary Figure 6: Dynamical autoencoder vs autoencoder. We provide a simplified figure to demonstrate the difference between our proposed model–dynamical autoencoder–and the standard autoencoder model respectively in subplots (a)(b). We highlight that our proposed model learns a distribution conditioned on system inputs and is better tailored for anomaly detection of dynamical systems.

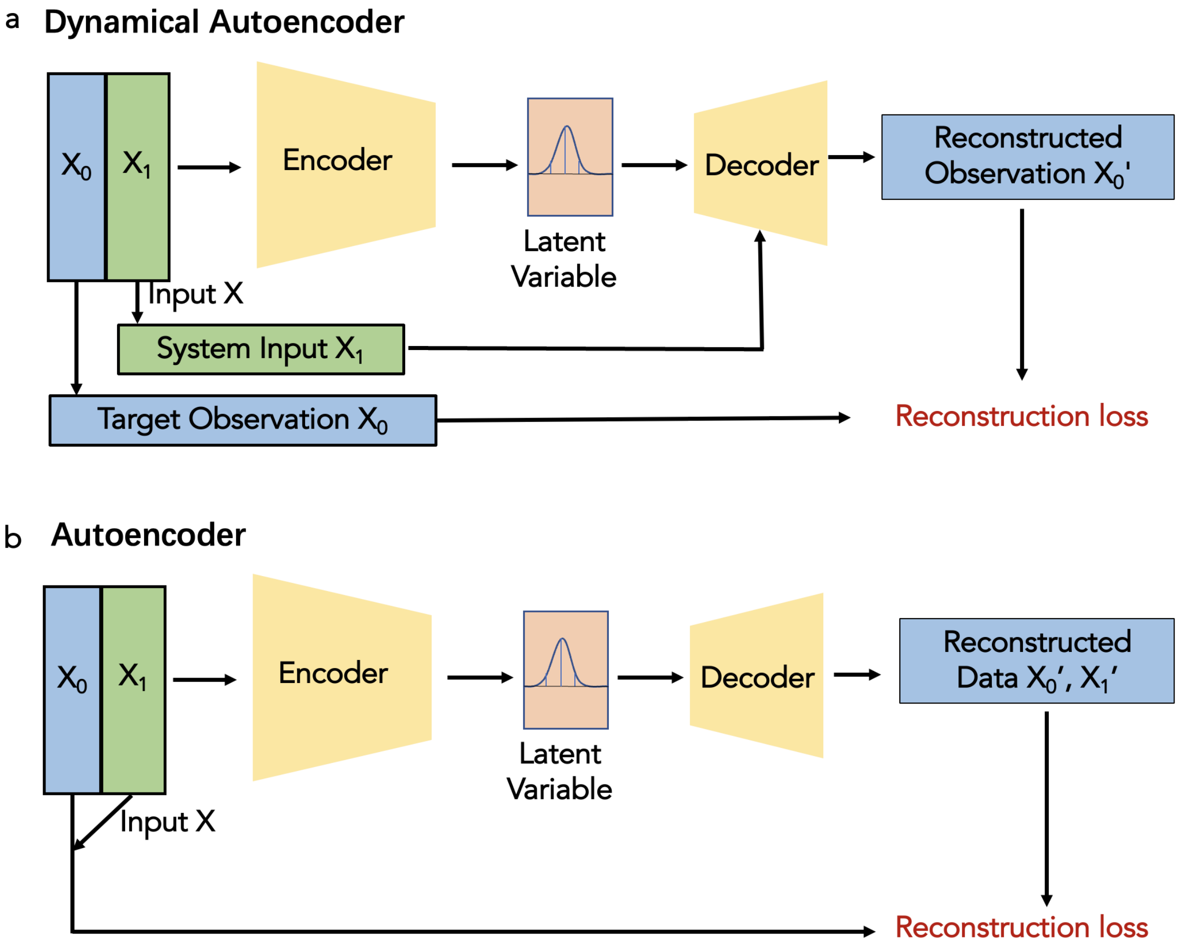

Supplementary Note 1: Mathematical Motivations for Dynamical Anomaly Detection

Hypothesis testing in dynamical systems We formulate hypothesis testing in dynamical systems and highlight its difference against the classical setup. For simplicity, we consider a discrete dynamical system with a fixed time length in a Euclidean space. A more generalized and formal study is left as future directions. In particular, consider a random mapping

$$f : \mathscr{X} \times \theta \times \mathscr{U} \to \mathscr{X},$$

where f describes the transition probability that maps an inner state $x_t \in \mathscr{X}$, a system input $u_t \in \mathscr{U}$, and a time-invariant system parameter $\theta \in \Theta$ to a **random** next state $x_{t+1} \in \mathscr{X}$. More formally, for $t = 1, 2, ..., T$,

$$x_{t+1} \sim f(x_t, \theta, u_t),$$

Furthermore, we consider the case when system inputs are sampled from a distribution that is independent from the system itself, $u_{1:T} \sim U$.

Our goal is to detect whether an observed sample comes from a normal system, where system parameters are sampled from the null hypothesis H_0, or from an abnormal system, where the parameters are sampled from the alternative hypothesis H_1:

$$\begin{aligned} H_0 &: \theta \sim \Theta_0, \\ H_1 &: \theta \sim \Theta_1. \end{aligned}$$

The above formulation subsumes many real-world problems. For example, if we aim to detect abnormal electric vehicle batteries, θ can describe battery health, whereas the signals x_t are recorded by the battery management system under the charging current u_t.

The benefit of viewing time series as observations from dynamical systems is that it turns the high dimensionality caused by the long time horizon into our favor. Intuitively, the problem dimension is determined by the dimension of the system dynamics θ, whereas additional observations x_t, u_t tell us more about the unobserved parameter θ. We pursue this idea in the next subsection.

Likelihood tests for the dynamical systems Following the notations above, the anomaly detection task can be stated via the optimization problem below:

$$\max_{f \in \mathscr{F}} \mathbb{E}_{u_{1:t} \sim U, \theta \sim \Theta_1} [\mathbb{I}\{f(x_{1:T}, u_{1:T}) = 1\}], \tag{1}$$

$$s.t \ \ \mathbb{E}_{u_{1:t} \sim U, \theta \sim \Theta_0} [\mathbb{I}\{f(x_{1:T}, u_{1:T}) = 1\}] \leq \alpha, \tag{2}$$

where $\mathbb{I}$ denotes the indicator function, the hypothesis class $\mathscr{F}$ is a subset of prediction functions $\{f : \mathscr{Y}^{\otimes T} \times \mathscr{U}^{\otimes T} \to \{0, 1\}\}$, and the parameter α controls the false discover rate. Here, the objective aims to maximize the power of the test on the alternative hypothesis, i.e., the percentage of anomaly detected. For now, we assumed that the alternative hypothesis is simple and single-valued.

With the above goal in mind, we then have for any α, the optimal solution to the optimization problem above (also known as the uniformly most powerful test) can be written as thresholding the conditional likelihood ratio below

$$f(x_{1:T}, u_{1:T}) = \mathbb{I}\left\{ \prod_t \frac{p_1(x_t | u_{t-1}, x_{t-1})}{p_0(x_t | u_{t-1}, x_{t-1})} > c \right\}, \tag{3}$$

where p_0 denotes the likelihood under the null hypothesis, p_1 denotes the likelihood under the alternative hypothesis, and c is chosen such that $E_{u_{1:t} \sim U, \theta \sim \Theta} [\mathbb{I}\{f(y_{1:T} | u_{1:T}) = 1\}] = \alpha$. The proof is a direct application of the famous Neyman-Pearson theorem.

The interesting observations are twofold. First, the optimal detector is independent of the input distribution $u_{1:t} \sim U$, but only depends on the conditional distribution $p(x_{1:T} | u_{1:T})$. Second, we get a product form that resembles

likelihood ratios for independent variables, which suggests that we may get stronger statistical significance from martingale-style concentration bounds.

In practice, very often the alternative hypothesis is composite instead of simple. In this setup, the uniformly most power test may not exist. Alternatively, we have the following guarantee on false discover rate. If for any y, x, u, there exists a θ such that $p_\theta(y|x,u) = 1$, then we have that

$$f(y_{1:T}, u_{1:T}) = \mathbb{I}\left\{\prod_t p_0(x_t|u_{t-1}, x_{t-1}) < c\right\},$$

is the generalized likelihood ratio test. Under the null hypothesis, the likelihood ratio within the indicator function converges in distribution to χ-squared distribution with freedom d, where d is the dimension of the parameter space. The proof applies Wilk's theorem to the following equation:

$$\sup_{p_1} \frac{p_1(x_{1:T}, u_{1:T})}{p_0(x_{1:T}, u_{1:T})} = \prod_t \frac{1}{p_0(x_t|u_{t-1}, x_{t-1})}.$$

The above proposition suggests that, asymptotically, we could simply reject the hypothesis by thresholding the likelihood according to the χ^2 test and control the false discovery rate. Since log-likelihood is monotonic in likelihood, it is equivalent to finding a classifier of the following form:

$$f(x_{1:T}, u_{1:T}) = \mathbb{I}\left\{l(\theta) + \sum_{t \le T} l(x_t|u_{t-1}, x_{t-1}, \theta) < c\right\}, \quad (4)$$

where we dropped the subscript and use $l(\theta, x, u) := \log p_0(\theta, x, u)$ to denote the log-likelihood under the distribution of the null hypothesis. The form in (4) motivates us to propose the anomaly detection algorithm in the next section.

DyAD: Auto-encoder-based anomaly detection model We have seen above that the key to anomaly detection via hypothesis testing is to learn the distribution of $\theta \sim \Theta_0$. We adopt the variational inference (as in variational autoencoder[2], diffusion models[3], etc.) formulation for this task. In particular, we parameterize the family of likelihoods $\mathscr{L}$ via weights in neural networks. Then we want to identify the likelihood $l^* \in \mathscr{L}$ that minimizes the KL divergence between the empirical distribution $\hat{p}_0$ and the probability function p_{l^*} induced by the learned likelihood l^*. For a more detailed discussion on the variational inference, we refer the readers to Section 2.2 in [2]. Hence, we get,

$$\min_{l \in \mathscr{L}} D_{KL}(\hat{p}_0, p_{l^*}) = \mathbb{E}_{\mathbf{x}_i, \mathbf{u}_i, \theta_i \sim \hat{p}_0}\left[\log\left(\frac{p_0(\mathbf{x}_i, \mathbf{u}_i, \theta_i)}{p_{l^*}(\mathbf{x}_i, \mathbf{u}_i, \theta_i)}\right)\right],$$

where $\mathbf{u}_i, \mathbf{x}_i$ are shorthands for the i_{th} sampled input and output sequences. We note that as the numerator is independent of l, we can equivalently solve

$$\max_{l \in \mathscr{L}} \mathbb{E}_{\mathbf{x}_i, \mathbf{u}_i, \theta_i \sim \mathscr{D}_{train}}\left[l(\theta^i) + \sum_{t \le T} l(x_t^i|u_{t-1}^i, x_{t-1}^i, \theta^i)\right],$$

where we used the fact that likelihood can be rewritten as products due to the Markovian structure.

However, the above problem cannot be solved, because in practice we can only observe the system inputs and outputs $\mathbf{u}_i, \mathbf{x}_i$, whereas the system parameter θ remains unknown. Hence, a natural fix is to infer θ from observed data.

$$\max_{(l_e, l_d) \in \mathscr{L}} \mathbb{E}_{\mathbf{x}_i, \mathbf{u}_i \sim \mathscr{D}_{train}} \mathbb{E}_{\theta_i' \sim l_e(\cdot|\mathbf{x}_i, \mathbf{u}_i)}\left[\sum_{t \le T} l_d(x_t^i|u_{t-1}^i, x_{t-1}^i, \theta_i')\right],$$

where we marginalize over the unobserved θ by learning a posterior distribution. In practice, we cannot optimize over the entire probability space. Hence we simplify the problem with the following approximations:

1. We assume the distribution of the system parameters θ can be reparameterized (e.g., through a neural network) as a multivariate Gaussian $\theta \sim \mathcal{N}(0, I)$.

2. We assume that x_t is Gaussian conditioned on u_{t-1}, x_{t-1}, θ, i.e.,

$$x_t \sim \mathcal{N}(\mu_d(u_{t-1}, x_{t-1}, \theta), \sigma_d^2 I),$$

   where μ_d is a function to be learned and σ is a hyper-parameter.

3. We assume the posterior is also Gaussian, i.e.,

$$\theta \sim \mathcal{N}(\mu_e(u_{t-1}, y_{t-1}), \sigma_e^2(u_{t-1}, y_{t-1})),$$

   where μ_e and σ are functions to be learned.

Putting everything together and use the ELBO (evidence lower bound) trick, we get something that is similar to the LSTM-autoencoder model, but with one key difference, that the system input is sent into the decoder. The optimization problem now becomes:

$$\min_{\mu_d, \mu_e, \sigma_e} \mathbb{E}_{\mathbf{x}_i, \mathbf{u}_i \sim \mathscr{D}_{train}, \theta \sim \mathcal{N}(\mu_e, \sigma_e^2 I)} \left[D_{KL}(\mathcal{N}(\mu_e, \sigma_e^2), \mathcal{N}(0, I)) + \sum_{t \leq T} (y_t^i - \mu_d)^2 / \sigma_d^2 \right]$$

where D_{KL} denotes the KL divergence, we take the negative sign and use that log-likelihood of Gaussian is quadratic. The notations μ_d, μ_e are parameterized by recurrent neural networks and are shorthands for

$$\begin{aligned} &\mu_e = \mu_e(\mathbf{x}_i, \mathbf{u}_i), \sigma_e^2 = \sigma_e^2(\mathbf{x}_i, \mathbf{u}_i), \\ &\mu_d = \mu_d(u_{t-1}^i, x_{t-1}^i, \theta). \end{aligned}$$

We further simplify the KL divergence using properties of Gaussian distributions and get

$$\min_{\mu_d, \mu_e, \sigma_e} \mathbb{E}_{\mathbf{x}_i, \mathbf{u}_i \sim \mathscr{D}_{train}, \theta \sim \mathcal{N}(\mu_e, \sigma_e^2 I)} \left[\underbrace{\|\mu_e\|^2 + tr(\sigma_e^2) - \log(|\sigma_e^2|)}_{\text{variational reg. loss}} + \underbrace{\sum_{t \leq T} (y_t^i - \mu_d)^2 / \sigma_d^2}_{\text{recon. loss}} \right] \tag{5}$$

If we view μ_e, σ_e as output of the encoder network and μ_d as output of the encoder network, then we can adopt the variational autoencoder training procedure with the modification specified in the Figure 2c. The key difference is that, instead of requiring the network to learn to retrieve all data dimensions, our model aims to learn the internal states θ of the system. Therefore, the decoder in DyAD, which now serves as a dynamical system, is responsible for simulating the system, and retrieving the response from the latent representation and the internal states. We note that some recent works (e.g.,[4,5]) also applied autoencoders to learning dynamical systems, yet our result provides the first derivation and application via anomaly detection.

Supplementary Note 2: Training details of methods

Data processing The three released datasets contain preprocessed data stored in the same format from hundreds of vehicles. Each piece of charging snippets contains current, minimum cell voltage, maximum cell voltage, total voltage, minimum sensor temperature, maximum sensor temperature and state-of-charge (SOC) information during EV charging periods. Each snippet has 128 timestamps sampled every 10 seconds (approximately 20 minutes of recording in total). The total voltage is scaled and shifted by a random float to normalize the value and to remove private information. Each data point is coupled with masked metadata on vehicle number, charging number, encoded mileage and timestamp.

The first dataset (Dataset Dahu) has 198 EVs in total, 30 of which are abnormal, and 470,000 charging snippets. The second dataset (Dataset Socea) has 49 EVs in total, 16 abnormal labels, and 195,000 charging snippets. The third dataset (Dataset Naobop) has 100 EVs in total, 9 abnormal labels, and 29,000 charging snippets. Each dataset contains data from the same vehicle model. Our model and baselines were trained and tested on each dataset separately. We report the average performance across three datasets.

Autoencoder (AE). As a popular algorithm in time series anomaly detection, an autoencoder (AE) can be used to detect abnormal events in a sequence by calculating the reconstruction error[6,7]. The input charging data are simply flatten and then an eight-layer AE is trained. The latent space is set to be 32. The network also includes batch-normalization and dropout layers. We use minibatch training with a batch size of 128 and the Adam optimizer with an initial learning rate of 0.001. Abnormal charging snippets are assumed to have higher reconstruction errors while normal snippets have lower values.

Graph deviation network (GDN). A recent graph-based algorithm that is specifically designed for multivariate time series anomaly detection, named graph deviation network (GDN)[8], is adapted to the battery fault detection task as a baseline. It utilizes graph attention layers to learn the inter-variable relationships. The Adam optimizer is used with a learning rate of 0.001 and the batch size is set to 128. The algorithm trains on a clean training set without anomalies so that only normal relationships between variables are learned. At the test stage, abnormal snippets with deviation from normal relationships are detected and thus produce a higher forecasting error. Other hyper-parameters are employed from the original GDN algorithm[8] except that the window size is set to be 32 with a stride of 16.

Support vector data description (SVDD). We implement the deep SVDD algorithm with an autoencoder as a feature extractor[9]. The algorithm aims to minimize the volume of a hypersphere that encloses representations of the normal data and forces the autoencoder to extract the common factors of variation. The architecture of the autoencoder is the same as the previous one. The batch size is set to be 64 and the Adam optimizer is used with a learning rate of 0.001 to train the model.

Gaussian process (GP). We implement the Gaussian process model using the open library in `https://github.com/Lleyton-Ariton/oddity`. More specifically, we treat each pieces of time series as sampled from a Gaussian process prior and infer the posterior mean. We can then compute the likelihood of observing the sample by computing its residual error against the posterior mean. We did not reweight the MSE error according to the covariance computed from a GP model. Our implementation of GP can be improved in multiple ways, such as using a learned kernel, or improving the sampling method of observations. We hope these ideas will be explored in future works.

Five fold evaluation. Recall that we have 292 normal and 55 abnormal vehicles. First, the normal and abnormal vehicles are randomly partitioned into five equal subgroups, denoted as $N_{1,2,3,4,5}$ (normal) and $A_{1,2,3,4,5}$ (abnormal). For each fold, four normal subgroups and one abnormal group are used for training and validation, and one normal subgroup and four abnormal groups are for test. For example, if the fold number is i, then we use $N_{\{1,2,3,4,5\}\setminus i}$ to train the model, $N_{\{1,2,3,4,5\}\setminus i} \cup A_i$ to tune the threshold τ and $N_i \cup A_{\{1,2,3,4,5\}\setminus i}$ to test the detection performance.

Supplementary Note 3: Additional works on deep-learning based anomaly detection Deep learning based anomaly detection algorithms started from detecting anomaly in image data. Some previous works achieved anomaly detection in feature space by forcing the feature concentration of normal samples[10,11]. Some works enhance the representation power of networks by introducing contrastive learning[12,13] and data transformation[14]. Recent works are more likely to introduce comprehensive deep learning techniques into anomaly detection. For example, self-supervised neural network and channel attention modules are both proposed for vision task[15]. A feature memory bank and nearest neighborhood search are employed for anomalies on industrial images[16]. A very thorough discussion of the-state-of-art algorithms for anomaly detection can be found in recent review articles[17,18], which indicates that current anomaly detection algorithms have mature and systematic development. As to the EV battery safety, as mentioned before, EV batteries with highly complex nonlinear characteristics would be difficult to conduct anomaly detection. Multivariate time series anomaly detection would be the most relevant deep learning topic to battery anomaly detection. For example, an LSTM-based encoder-decoder network and reconstruction errors are used to model the time series reconstruction probabilities[19]. Multivariate correlations are captured by considering each univariate series as an individual feature and including two graph attention layers to learn the dependencies of multivariate series in both temporal and feature dimensions[20]. Graph neural networks are adopted to learn the inter-variable interactions[8]. However, battery anomalies are always marked on the vehicle level rather than the event level, which requires the mentioned multivariate time series anomaly detection algorithms to be redesigned.

Supplementary References

1. Lu, Y. *et al.* A method of cell-to-cell variation evaluation for battery packs in electric vehicles with charging cloud data. *eTransportation* **6**, 100077 (2020).
2. Kingma, D. P. & Welling, M. Auto-encoding variational bayes. *arXiv preprint arXiv:1312.6114* (2013).
3. Ho, J., Jain, A. & Abbeel, P. Denoising diffusion probabilistic models. *Adv. Neural Inf. Process. Syst.* **33**, 6840–6851 (2020).
4. Girin, L. *et al.* Dynamical variational autoencoders: A comprehensive review. *arXiv preprint arXiv:2008.12595* (2020).
5. Mehta, V. *et al.* Neural dynamical systems: Balancing structure and flexibility in physical prediction. In *2021 60th IEEE Conference on Decision and Control (CDC)*, 3735–3742 (IEEE, 2021).
6. Aggarwal, C. C. *Outlier Analysis* (Springer, 2013).
7. Baur, C., Wiestler, B., Albarqouni, S. & Navab, N. Deep autoencoding models for unsupervised anomaly segmentation in brain mr images. In *International MICCAI brainlesion workshop*, 161–169 (Springer, 2018).
8. Deng, A. & Hooi, B. Graph neural network-based anomaly detection in multivariate time series. In *Thirty-Fifth AAAI Conference on Artificial Intelligence*, 4027–4035 (AAAI Press, 2021).
9. Ruff, L. *et al.* Deep one-class classification. In Dy, J. G. & Krause, A. (eds.) *Proceedings of the 35th International Conference on Machine Learning*, vol. 80 of *Proceedings of Machine Learning Research*, 4390–4399 (PMLR, 2018).
10. Schölkopf, B., Williamson, R. C., Smola, A. J., Shawe-Taylor, J. & Platt, J. C. Support vector method for novelty detection. In *NeurIPS 1999* (1999).
11. Lee, K., Lee, K., Lee, H. & Shin, J. A simple unified framework for detecting out-of-distribution samples and adversarial attacks. In *NeurIPS 2018* (2018).
12. Winkens, J. *et al.* Contrastive training for improved out-of-distribution detection. *arXiv preprint arXiv:2007.05566* (2020).
13. Tack, J., Mo, S., Jeong, J. & Shin, J. CSI: novelty detection via contrastive learning on distributionally shifted instances. In Larochelle, H., Ranzato, M., Hadsell, R., Balcan, M. & Lin, H. (eds.) *Advances in Neural*

Information Processing Systems 33: Annual Conference on Neural Information Processing Systems 2020, NeurIPS 2020, December 6-12, 2020, virtual (2020).

14. Golan, I. & El-Yaniv, R. Deep anomaly detection using geometric transformations. In *NeurIPS 2018* (2018).

15. Ristea, N.-C. *et al.* Self-supervised predictive convolutional attentive block for anomaly detection. In *Proceedings of the IEEE/CVF Conference on Computer Vision and Pattern Recognition (CVPR)*, 13576–13586 (2022).

16. Roth, K. *et al.* Towards total recall in industrial anomaly detection. In *Proceedings of the IEEE/CVF Conference on Computer Vision and Pattern Recognition (CVPR)*, 14318–14328 (2022).

17. Bergmann, P., Fauser, M., Sattlegger, D. & Steger, C. Mvtec ad–a comprehensive real-world dataset for unsupervised anomaly detection. In *Proceedings of the IEEE/CVF conference on computer vision and pattern recognition*, 9592–9600 (2019).

18. Yang, J. *et al.* Openood: Benchmarking generalized out-of-distribution detection. *arXiv preprint arXiv:2210.07242* (2022).

19. Malhotra, P. *et al.* Lstm-based encoder-decoder for multi-sensor anomaly detection. *CoRR* **abs/1607.00148** (2016). 1607.00148.

20. Zhao, H. *et al.* Multivariate time-series anomaly detection via graph attention network. In Plant, C., Wang, H., Cuzzocrea, A., Zaniolo, C. & Wu, X. (eds.) *20th IEEE International Conference on Data Mining, ICDM 2020, Sorrento, Italy, November 17-20, 2020*, 841–850, DOI: 10.1109/ICDM50108.2020.00093 (IEEE, 2020).
